# A Narrative Review on the Impact of Probiotic Supplementation on Muscle Development, Metabolic Regulation, and Fiber Traits Related to Meat Quality in Broiler Chickens

**DOI:** 10.3390/microorganisms13040784

**Published:** 2025-03-28

**Authors:** Robert Herich, Renáta Szabóová, Viera Karaffová, Maria Paula Racines, Miroslava Anna Šefcová, Marco Larrea-Álvarez

**Affiliations:** 1 Department of Morphological Disciplines, University of Veterinary Medicine and Pharmacy in Košice, 041 81 Košice, Slovakiaviera.karaffova@uvlf.sk (V.K.); 2 Department of Biology and Physiology, University of Veterinary Medicine and Pharmacy in Košice, 041 81 Košice, Slovakia; 3 Facultad de Ciencias de la Salud, Carrera de Medicina, Universidad Espíritu Santo, Samborondón 092301, Ecuador

**Keywords:** broiler chickens, probiotics, gut–muscle axis, skeletal muscle, muscle fiber type, meat quality parameters, performance

## Abstract

Public concern over drug resistance has led to governmental regulations banning the use of antibiotics as growth promoters, stimulating interest in developing complementary strategies to maintain animal production, mitigate infections, and enhance muscle characteristics and quality parameters, especially in meat-producing animals. Probiotics are recognized as a potential strategy for improving growth, primarily by promoting intestinal homeostasis. These microorganisms are suggested to modulate gut microbiota, preserving their ecosystem and influencing secondary metabolite production, which can directly or indirectly regulate skeletal muscle metabolism by influencing the expression of key muscle-related genes and the activity of various signaling factors. Several studies have documented the potential benefits of various strains of *Bacillus*, *Enterococcus*, and members of the Lactobacillaceae family on muscle characteristics. These studies have shown that probiotics not only modulated myogenic factors but also influenced proteins and enzymes involved in signaling pathways related to carbon metabolism, inflammatory response, mitochondrial dynamics, and antioxidant activity. These effects have been associated with improvements in meat quality parameters and enhanced growth performance. This manuscript seeks to present a brief overview of the impact of probiotic supplementation on muscle health and the quality of meat in broiler chickens.

## 1. Introduction

The population of farm animals has increased substantially in recent decades to meet the rising demand for meat. Particular management practices, improvements in nutrition, and genetic selection have allowed for enhancements in both growth and meat yields [1,2]. Meat quality encompasses various characteristics that determine its cost and marketability, including color, pH, texture, flavor, and water-holding capacity [3]. These attributes are influenced not only by the arrangement of different muscle fibers but also by the intramuscular fat (IMF) content [4]. Muscle fibers can be classified into oxidative (Type I and Type IIA) and glycolytic (Types IIX and IIB) fibers [5,6]. Type I fibers are associated with high-quality meat, appearing bright red, having small diameters, and reduced cross-sectional area, which enhances tenderness and reduces shear force. Furthermore, their metabolism does not impact pH and water retention, while their phospholipid content contributes to producing a strong and fragrant flavor [5,7,8,9].

Muscle tissue derives from the embryonic mesoderm, where progenitor cells proliferate and differentiate into somites. These structures later form the myotome, which houses muscle precursors called myoblasts, proliferating extensively before fusing into primary myofibers, a process controlled by the precise temporal expression of myogenic regulatory factors (MRF) [10,11,12]. During embryonic development, muscle grows through hyperplasia (increased cell number), while post-hatch growth relies on hypertrophy (fiber enlargement) via protein and myonuclei accretion [10,11,12,13,14]. Satellite cells, the main source of new myonuclei, remain quiescent in adults beneath the basement membrane. Upon activation, they differentiate and fuse with myofibers, increasing fiber diameter under the regulated expression of MRFs [15,16,17]. The activation, stability, and transcriptional activity of these factors are regulated by various protein kinases and signaling molecules; they help regulate the processes that allow muscle cells to mature and merge. Moreover, various non-coding RNAs have been observed to regulate skeletal muscle development in chickens by modulating the expression of factors during proliferation and differentiation [18].

It has been suggested that both fiber size and type composition can adjust in response to changes in muscle function [19,20,21]. For example, muscle fiber transformation and IMF deposition have been suggested to be influenced by the gut microbiome [22,23,24]. Indeed, the concept of a gut–muscle axis has gained significant consideration, as it focuses on the effects of gut microbiota on skeletal muscle function and metabolism [25,26]. Consequently, various strategies have been proposed for enhancing meat quality by influencing the gut microbiota, including fecal transplantation, dietary component administration, and probiotic supplementation [27,28,29]. Probiotics are well known for their impact on the small intestine with regard to architecture, integrity of the epithelium, microbiota composition, and inflammation [30,31,32,33,34,35]. Also, they are associated with the production of short-chain fatty acids (SCFAs), secondary bile acids (SBAs), and branched-chain amino acids (BCAAs), which are known to contribute to gut health and metabolism [25,36,37].

Short-chain fatty acids are absorbed by specialized transporters and recognized by various receptors expressed in adipose tissue, neurons, immune cells, heart, and skeletal muscle [38,39,40,41,42]. Signaling through these receptors can ultimately modify gene expression via intracellular messengers [43,44,45]. SCFAs can interact with deacetylases and acetyl transferases, promoting gene transcription [46,47,48] while also being utilized as a substrate for β-oxidation in the mitochondria [49]. The presence of these metabolites is known to contribute to the formation of oxidative fibers [50]. Furthermore, SCFAs have been observed to indirectly affect skeletal muscle. These metabolites stimulate the secretion of hormones by enteroendocrine cells, which are associated not only with glucose absorption and metabolism but also with enhanced recruitment of skeletal muscle microvasculature [51]. Compounds released from the metabolism of bile acids and amino acids can also impact muscle metabolism [52]. Secondary bile salts, for instance, can activate cellular receptors involved in cell growth and differentiation and turn on genes associated with skeletal muscle phenotype [53]. Similarly, branched-chain amino acids (BCAAs) can serve not only as nutrients but also as signaling molecules that influence skeletal muscle development [54,55,56]. Probiotics and probiotic-derived metabolites are known for their immunomodulatory properties, particularly their ability to downregulate the expression of inflammatory cytokines. This could potentially mitigate the harmful effects of inflammation on skeletal muscle [57,58] as it has been linked to alterations in muscle protein balance and mitochondrial metabolism, leading to atrophy [59].

Probiotics are products that contain live microorganisms, which are expected to provide benefits to the host when consumed in sufficient amounts [60]. These include well-known lactic acid bacteria, bifidobacteria, or yeasts [61]. The unique structural features and ecological roles of probiotics are essential for their positive impact on the host. For instance, these bacteria produce peptides with bactericidal effects (bacteriocins) and organic acids synthesized during fermentation, creating an acidic environment that inhibits the growth of invading microorganisms [62,63,64]. Furthermore, probiotic bacteria can adhere to host tissue through specific and non-specific interactions, directly competing for nutrients and niche colonization with potential pathogens [65,66]. These beneficial microorganisms can also interact with pattern recognition receptors on host cells, which detect specific molecular patterns present in bacterial cell walls, such as lipoproteins, teichoic acids, peptidoglycan, and capsule polysaccharides [67]. Activation of these receptors initiates a signaling cascade that modulates the expression of genes involved not only in immune modulation but also in cell survival and proliferation [58,68]. The use of these bacteria in poultry has been effective in maintaining the epithelial barrier and enhancing intestinal conditions in terms of architecture and mucus production [69,70,71]. In general, the administration of probiotics has been shown to enhance performance parameters such as body weight, feed intake, and feed conversion ratio in broilers [72,73].

The potential of modulating the host ecosystem and influencing muscle characteristics presents exciting opportunities for improving meat quality parameters through the use of feed additives, including probiotics, which have been reported to affect muscle metabolism and phenotype in various animals [25,36,74,75,76,77]. In broiler husbandry, probiotic supplementation has proved to influence the expression of myogenic factors alongside genes encoding proteins and enzymes involved in signaling pathways, carbon utilization, immune response, mitochondrial dynamics, and oxidative balance. The modulation of these factors has been associated with enhancements in meat quality traits, including pH, color, water-holding capacity, and shear force, along with improved growth performance.

This manuscript aims to summarize the findings that demonstrate the beneficial effects of probiotics regarding muscle development, metabolic regulation, and fiber traits related to meat quality in broiler chickens. To create this narrative review, searches were conducted using PubMed, Scopus, Web of Science, and Google search engines. Articles published in English from 2010 onwards were identified using the following terms: “probiotics” AND “broiler chickens” AND “muscle phenotype”; “probiotic supplementation” AND “myofiber structure” AND “broilers”; “broiler chickens” AND “myogenic factors” AND “probiotics”; “gut microbiota” AND “muscle metabolism” AND “broiler chickens” AND “meat quality”. Two researchers independently searched the specified keywords, with duplicate documents being discarded. We included experimental peer-reviewed studies reporting data on myofiber structure, myogenic factors, oxidative metabolism, signaling pathways (AMPK, PGC-1α), and carbon metabolism. Studies had to involve broiler chickens and provide measurable outcomes related to muscle characteristics, metabolism, and, if available, meat quality parameters.

## 2. Muscle Fibers and Meat Quality

In broiler chickens, skeletal muscle constitutes the majority of body mass, making its growth and efficiency critical for meat production. Muscle size and mass are influenced by a combination of genetic predisposition, environmental conditions, nutritional intake, and overall health status. These factors are orchestrated by intricate molecular mechanisms that govern muscle development and regulation [78,79,80].

Myoblasts undergo extensive proliferation and subsequently fuse to form primary myofibers, a process governed by the tightly regulated temporal expression of MRFs, including myogenic factor 5 (MYF5), myoblast determination protein 1 (MYOD), myogenin (MYOG), and myogenic regulatory factor 4 (MRF4). These primary fibers serve as a scaffold for the formation of secondary fibers. During embryonic development, muscle growth is primarily driven by hyperplasia, characterized by an increase in muscle cell number, whereas post-hatch growth is dominated by hypertrophy, involving the enlargement of existing fibers [10,11,12]. This muscle hypertrophy occurs through protein and myonuclei accretion into pre-existing myofibers [13,14]. Satellite cells serve as the primary source of these new myonuclei. These cells remain quiescent in adult animals and reside in a niche adjacent to existing myofibers [81,82]. Once activated and differentiated, satellite cells fuse with myofibers, contributing to an increase in muscle fiber diameter. In the quiescent state, satellite cells express factors such as *MYF5*, *MYOD*, and Paired box protein 7 (*PAX7*). The downregulation of *PAX7* and the upregulation of *MYOG* are characteristic of early differentiation, while the expression of myosin heavy chain is a hallmark of fully differentiated cells. Also, myocyte enhancer factors (MEF2A, MEF2D) are known to regulate muscle gene expression [15,16,17]. The processes involving the development of skeletal muscle are tightly regulated not only by the aforementioned factors but also by others associated with the PAX and Sine Oculis-Related Homeobox (Six) families [83,84,85].

The activation, stability, and transcriptional activity of MRFs are regulated by various protein kinases. During the early stages, protein kinase B (PKB), also known as Akt1, protein kinase A (PKA), cyclin-dependent kinases (CDK2, CDK4), and the fibroblast growth factor receptor (FGFR) are essential for myoblast proliferation [18,86]. During the middle stages of myocyte differentiation and fusion, several signaling pathways and enzymes work together, including the insulin-like growth factor receptor (IGFR)-mitogen-activated protein kinase (MAPK) pathway, glycogen synthase kinase 3 β (GSK3β), CDK5, CDK9, and the transforming growth factor-β activated Kinase 1 (TAK1)-MKK3/6-p38α pathway. These molecules help regulate the processes that allow muscle cells to mature and merge. The final stages of hypertrophy and myotube formation are regulated by the mechanistic target of rapamycin (mTOR), ribosomal protein S6 kinase (S6K), and ribosomal S6 kinase 2 (RSK2) [18,86]. Moreover, various non-coding RNAs, including microRNAs (miRNAs), long non-coding RNAs (lncRNAs), and circular RNAs (circRNAs), have been shown to influence skeletal muscle development in chickens by modulating the expression of key factors during proliferation and differentiation [18].

**Figure 1:**
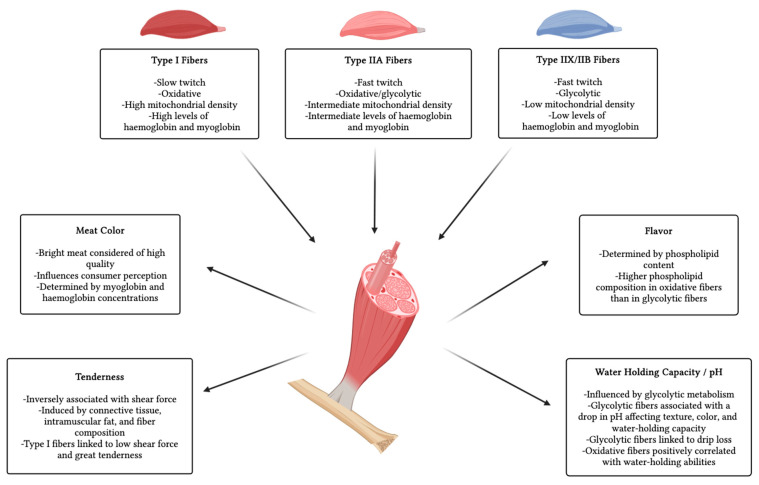
Muscle characteristics and the quality of meat. The type, size, and quantity of muscle fibers, along with the intramuscular fat (IMF) content, are crucial for determining essential parameters that affect meat quality and marketability, including meat color, tenderness, flavor, and pH levels. Created in BioRender. Larrea-Álvarez, M. (2025) https://BioRender.com/bakknd0 (accessed on 15 March 2025); Agreement number RL282T7NHQ).

The primary components of skeletal muscle are fibers with specific metabolic and morphological characteristics, classified according to various criteria. For instance, muscles are categorized by contraction speed into fast twitch and slow twitch fibers. Alternatively, fibers can be classified based on their metabolic properties into oxidative and glycolytic fibers. In general, three main types are recognized: Type I, or slow-twitch oxidative fibers; type IIA, or fast-twitch oxidative fibers; and types IIX and IIB, or fast-twitch glycolytic fibers. Slow-twitch fibers are associated with myosin heavy chain I (MyHC I) isoforms, which exhibit lower ATPase activity and shorter lever arms compared to Myosin Heavy Chain II (MyHC II) isoforms found in fast-twitch fibers [5,6]. The arrangement of different muscle fibers, including their type, size, quantity, and intramuscular fat content, influences meat quality [4]. Meat quality refers to the attributes that determine its value and marketability. Key characteristics associated with meat quality include color, pH, texture, flavor, and water-holding capacity [3] (Figure 1). For example, the concentration of hemoglobin and myoglobin found in muscle fibers determines meat color. Oxidative fibers, which have higher levels of these proteins, appear bright red. In contrast, glycolytic fibers, with lower myoglobin concentrations, have a pale color [5]. Meat color indicates muscle fiber types, influencing texture, flavor, and overall eating experience, and it is also associated with consumer perception and buying decisions, with bright meat considered fresh and high-quality [87]. Significantly low pH levels negatively affect muscle protein stability, leading to a loss of bright color, resulting in a paler appearance, reduced water retention capacity, and a soft texture [8].

Tenderness is an essential property associated with meat quality, measured by assessing shear force, with lower force indicating greater tenderness. This characteristic is influenced by connective tissue, intramuscular fat, and fiber composition [88,89]. For instance, oxidative fibers have smaller diameters and a reduced cross-sectional area compared to glycolytic fibers. A high proportion of type IIB fibers is linked to reduced tenderness and increased shear force, whereas type I fibers are associated with greater tenderness and lower shear force [9,90]. Another factor influencing meat quality is pH levels, which are affected by the presence of glycolytic fibers, ATP, and glycogen. Glycolytic fibers, which rely heavily on glycogen and ATP for energy, can lead to a rapid drop in pH post-mortem, resulting in meat with a softer texture, paler color, and reduced water-holding capacity. In contrast, oxidative fibers maintain a higher pH and better protein integrity, which aids in retaining more water [8]. Furthermore, the flavor of meat is influenced by its phospholipid composition, as these lipids contribute to the development of flavor compounds during cooking. Oxidative fibers have a higher phospholipid content than glycolytic fibers and, thus, significantly contribute to producing a strong and fragrant flavor [7,91] (Figure 1).

As mentioned, two phases are identified in skeletal muscle development: hyperplasia and hypertrophy. While hyperplasia is established during embryogenesis, it has been suggested that both fiber size and type composition can adapt to changes in muscle function [19,20,21]. Environmental factors such as temperature or gut microbiota can induce fiber transformation from type IIB to type I, or vice versa, in various farm animals [92,93]. These phenotypic transformations are governed by diverse pathways and their corresponding genes, influenced by factors such as the animal’s age and nutritional status [19,86].

The shift from type II to type I fibers has been linked to the stimulation of the AMP-activated kinase (AMPK), which influences mitochondrial biogenesis and induces an overall shift toward an ATP-producing catabolic state, easing the transformation from fast-twitch to slow-twitch fibers [94,95]. In fact, this kinase also regulates the expression of the peroxisome proliferator-activated receptor gamma coactivator 1-α (PGC-1α), which contributes to maintaining mitochondrial oxidative metabolism. It exerts its effects by interacting with factors that impact the quantity and size of oxidative fibers, such as the peroxisome proliferator-activated receptor α (PPARα) [96,97]. PGC-1α also interacts with sirtuin factors (SIRT1, SIRT3) that regulate mitochondrial function and oxidative metabolism. In addition, the participation of the forkhead box O (FOXO), Wingless/Integrates (Wnt), sonic hedgehog (Shh), and calcium ion (Ca^2+^) pathways is also key for regulating fiber transition [98,99,100,101,102]. Stimulation of these pathways leads to the activation of transcription factors that regulate the expression of genes associated with the oxidative phenotype [103,104].

In particular, the gut microbiome has been suggested as a key factor in regulating the transformation of muscle fibers [22,23,24]. The concept of a gut–muscle axis, focusing on how interactions between gut microbiota and the host affect skeletal muscle function and metabolism, has gained attention [25,26]. Indeed, treatment with probiotics has been tested in healthy rodents and aging or cachectic mice to determine their effects on skeletal muscle function and performance. For example, supplementation of *Lactiplantibacillus plantarum* TWK10 to ICR mice not only improved the mass of the gastrocnemius but also resulted in a higher proportion of type I fibers compared to control conditions. Furthermore, this probiotic strain also enhanced forelimb strength and swimming performance while reducing post-exercise levels of fatigue markers, including lactate, ammonia, and creatine kinase (CK) in plasma [105]. Similarly, grip strength was enhanced after administration of heat-killed *Bifidobacterium brevis* in Sprague Dawley rats and C57BL/6J mice. Probiotic treatment not only stimulated the activation of components of the mTOR pathway but also increased the expression of PGC1-α [106].

Treatment with *Lacticaseibacillus paracasei* PS23 demonstrated an attenuation of age-related muscle mass loss, increased grip strength, and improved muscle endurance in SAMP8 mice. These outcomes were attributed not only to increased mitochondrial biogenesis, oxygen consumption, and the expression of antioxidant enzymes in the muscle but also to a reduction in oxidative markers. Furthermore, probiotic exposure reduced the expression of proinflammatory cytokines while enhancing those linked to the anti-inflammatory response [107]. Another strain of *Lacticaseibacillus*, *L. casei* LC122 has been tested in older C57BL/6 mice and was found to improve grip strength and enhance the activity of antioxidant enzymes. Additionally, the same study showed that *B. longum* reduced muscle inflammation by modulating the expression of inflammatory cytokines, including IL-6, TNFα, and IL1β [108]. *Limosilactobacillus reuteri* (ATCC-PTA-6475) was also demonstrated to limit muscle loss as it increased the muscle fiber cross-sectional area of the gastrocnemius and reduced lesions associated with fiber infiltration and fibrosis in C57BL/6 ApcMIN leukemic and cachectic mice [109]. In cachectic adenocarcinoma BALB/c mice, administration of a mixture containing *L. plantarum* CJ LP133, *Leuconostoc mesenteroides* CJ LM119, and lyophilized kimchi, a Korean dish made of salted and fermented vegetables, resulted in the reduced expression of factors associated with the inflammatory response (IL-6) and atrophy markers (Atrogin-1) in muscle. These results were accompanied by an increase in PGC-1α expression and overall leg muscle mass [110]. Undoubtedly, modulating the gut microbiota to influence myofiber type conversion holds promise for enhancing meat quality and increasing yield in farm animals. Probiotics, in particular, are acknowledged for their wide-ranging beneficial effects, which largely depend on the bacterial strain and dosage. These microorganisms have not only been tested for improving intestinal and immune conditions but also for their ability to enhance performance and meat quality parameters in poultry.

## 3. Characteristics of Probiotics

**Figure 2:**
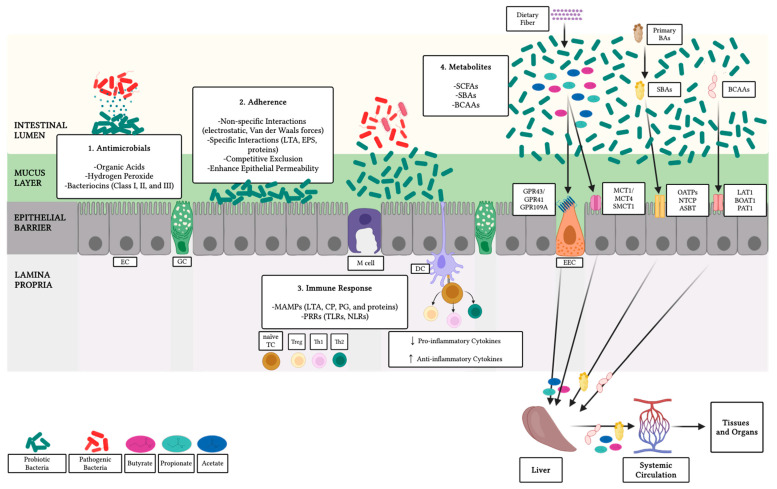
Interaction between probiotics, their products, and the host involves several mechanisms. The benefits associated with probiotic bacteria derive from their structural characteristics and ecological roles. The production of small organic acids and bacteriocins inhibits the growth of potential pathogens, while adherence to host tissues helps competitively exclude unwanted microorganisms and strengthen epithelial barrier function. Pattern recognition receptors on host cells identify microorganism-associated molecular patterns, triggering a signaling cascade that regulates the expression of inflammatory proteins, thereby influencing the immune response. Non-digestible fiber is metabolized by gut microbes into short-chain fatty acids that are recognized by cell receptors, absorbed through transporters, further metabolized, and transported to the liver, with some ultimately entering systemic circulation. Similarly, secondary bile acids and branched-chain amino acids, also produced by certain beneficial microbes, follow a similar path and are delivered to tissues and organs. LTA: Lipoteichoic Acid; EPS: Exopolysaccharides; MAMPs: Microbe-Associated Molecular Patterns, CP: Capsular Polysaccharide; PG: Peptidoglycan; PRRs: Pattern Recognition Receptors; TLRs: Toll-Like Receptors; NLRs: NOD-Like Receptors (Nucleotide-binding oligomerization domain-like receptors); TC: T Cell; Treg: Regulatory T Cell; Th1: T Helper 1 Cell; Th2: T Helper 2 Cell; SCFAs: Short-Chain Fatty Acids; SBAs: Secondary Bile Acids; BCAAs: Branched-Chain Amino Acids; EC: Enterocyte Cell; EEC: Enteroendocrine Cell; GC: Goblet Cell; DC: Dendritic Cell; GPR: G Protein-Coupled Receptor; MCT: Monocarboxylate Transporter; SMCT: Sodium-Coupled Monocarboxylate Transporter; OATPs: Organic Anion Transporting Polypeptides; NTCP: Sodium Taurocholate Co-transporting Polypeptide; ASBT: Apical Sodium-dependent Bile Acid Transporter; LAT: L-Type Amino Acid Transporter; BOAT: Bile Acid-CoA: Amino Acid N-Acyltransferase; PAT: Proton-Assisted Amino Acid Transporter. Created in BioRender. Larrea-Álvarez, M. (2025) https://BioRender.com/bakknd0 (accessed on 15 March 2025); Agreement number TA282T7NOG).

The benefits of probiotics are associated with their unique structural characteristics and ecological functions. Their contact enhances the integrity of the intestinal barrier, promotes microbiota diversity, and helps exclude potential pathogens. Furthermore, probiotics can stimulate the immune response by interacting with receptors that trigger the expression of cytokines (Figure 2) [66,111].

Antimicrobial metabolites fall into two main categories: proteins and small-molecular-mass organic acids. The latter exhibit a broad range of activity and primarily comprise organic acids generated during carbohydrate fermentation. These molecules not only reduce pH to create an acidic environment that is unfavorable for the growth of pathogens but also can directly disrupt bacterial membranes and interfere with their metabolism. Furthermore, some probiotics produce other antimicrobial compounds, including hydrogen peroxide and carbon dioxide [63,64]. Bacteriocins are cationic and hydrophobic peptides with bactericidal properties. The modification, export, and regulation of these proteins are closely controlled by the expression of multiple genes, including those responsible for producing self-immunity proteins [62]. Bacteriocins are released from cells and can not only destabilize the membrane but also disrupt gene expression and protein synthesis, leading to the death of both related and unrelated species [62,112]. These proteins are categorized into three groups. Class I bacteriocins, also known as lantibiotics, undergo posttranslational modifications, particularly forming lanthionine and/or methyllanthionine. These bacteriocins function by inhibiting bacterial cell wall synthesis and creating pores in membranes. Class II bacteriocins or non-lantibiotics open channels to allow for ion diffusion and membrane potential loss. Meanwhile, class III bacteriocins not only promote the hydrolysis of cell walls but also interfere with glucose uptake, as well as DNA and protein synthesis [113,114].

Adherence to the host tissue is seen as a vital factor for probiotics to confer benefits. These mechanisms encompass non-specific interactions, driven by electrostatic or van der Waals forces between bacterial cells and the mucosal surface, as well as specific interactions between particular molecules and their receptors on host cells [66]. LTAs, exopolysaccharides, and various proteins such as lectins, carbohydrate-binding proteins, fimbriae, and flagella have been shown to play a role in bacterial adhesion to epithelial surfaces [115,116,117,118,119,120]. Adhering to host tissue enables probiotic bacteria to directly compete with potentially harmful microorganisms. This competitive exclusion relies on bacterial interactions driven by nutrient competition and colonization of specific niches [65,121].

Probiotics can interact with immune system factors and regulate their responses. Components of bacterial cell walls, such as peptidoglycan, teichoic acids, capsule polysaccharides, and lipoproteins, contain specific molecular patterns recognized by receptors on host cells [67]. Among these, toll-like receptors (TLRs), produced by fibroblasts, leukocytes, and both epithelial and endothelial cells, recognize teichoic acids. Upon activation, they initiate an intracellular signaling cascade that regulates the expression of genes related not only to cytokine production but also to cell survival and proliferation. Peptidoglycan, conversely, is recognized by a different type of receptor called nucleotide-binding oligomerization domain-like receptors (NLRs), which also recruit proteins involved in signaling pathways. These pathways encompass the activation of various kinases, ultimately leading to the activation of the nuclear factor kappa-light-chain-enhancer of activated B cells (NF-κB) complexes [67,68]. As previously noted, probiotics are well-known for their immunomodulatory properties, as they can alter the production of both inflammatory and anti-inflammatory cytokines [122,123]. In farm animals, they have been demonstrated to not only increase the production of IL-4, IL-13, and IL-10 while reducing expression of IL-17 and interferon-γ (IFN-γ) but also to augment antibody levels [30,31,32,33,34,35]. Moreover, LTA-induced signaling has been observed to strengthen the expression of tight junction complexes [58].

Probiotic administration has been shown to improve performance parameters, including body weight, feed conversion ratio, and feed intake in poultry, especially in broiler chickens [30,72,73,124]. Probiotic treatment has also been linked to enhanced gut health, which is essential for maintaining the epithelial barrier. This barrier not only prevents the entry of invading microorganisms but is also crucial for nutrient absorption and host immunity [69,70,71]. Particularly, probiotics have been demonstrated to improve the architecture of the small intestine, as indicated by changes in the villus height to crypt depth (VH:CD) ratio, as well as increased goblet cell density and Muc2 production. The presence of these bacteria not only decreases enterobacteria counts and increases the number of Lactobacilli but also alleviates the negative effects induced by pathogens such as *Salmonella*, *Campylobacter*, *Clostridium*, or *Escherichia* [30,31]. Several bacterial species within the Lactobacillaceae family have demonstrated beneficial effects on broiler chicken performance, gut morphology, and immune function, including *Lactobacillus rhamnosus*, *L. paracasei* [72], *Ligilactobacillus salivarius* [125], *Lactobacillus acidophilus* [126], *Limosilactobacillus fermentum* [127,128,129,130], and *L. plantarum* [131]. Moreover, *Enterococcus faecium*, *Bacillus subtilis*, and *Clostridium butyricum* have been extensively studied as feed additives due to their ability to enhance growth performance, intestinal health, and meat quality [132,133,134]. Furthermore, the fungus *Aspergillus awamori* has been recognized for its role in mitigating oxidative stress, strengthening immune responses, and promoting growth [135]. Microbial metabolites, such as SCFAs, SBAs, and BCAAs, have been hypothesized to influence skeletal muscle metabolism and function [25,36,42,136,137]. SCFAs are primarily produced in the ileum and colon through anaerobic fermentation of non-digestible fiber by microbes in the gut lumen [37]. They produce hydrolases that degrade complex substrates, which are then utilized in fermentation reactions to generate pyruvate. Subsequently, pyruvate is converted into acetate, propionate, and butyrate by specific kinases [138]. The main SCFAs absorbed by epithelial cells are metabolized, utilized as energy, or transported to the liver via the portal vein. In the liver, propionate, for example, is used in gluconeogenesis pathways. In addition, SCFAs can reach the skeletal muscle, with those produced in the hindgut able to enter systemic circulation through the ileac vein, bypassing the liver and reaching skeletal muscle directly [25,36,37]. SBAs and BCAAs are absorbed by intestinal cells and can ultimately reach systemic circulation via the liver (Figure 2). Consequently, probiotics have been investigated for their potential role in regulating processes related to muscle development in both model and farm animals, as they have not only been shown to directly synthesize SCFAs or enhance their production by modulating gut microbiota composition and activity [139,140], but also have been linked to the production of SBAs and BCAAs [141,142].

## 4. Gut-Derived Secondary Metabolites and Muscle Cells

### 4.1. Direct Effects on Skeletal Muscle

**Figure 3:**
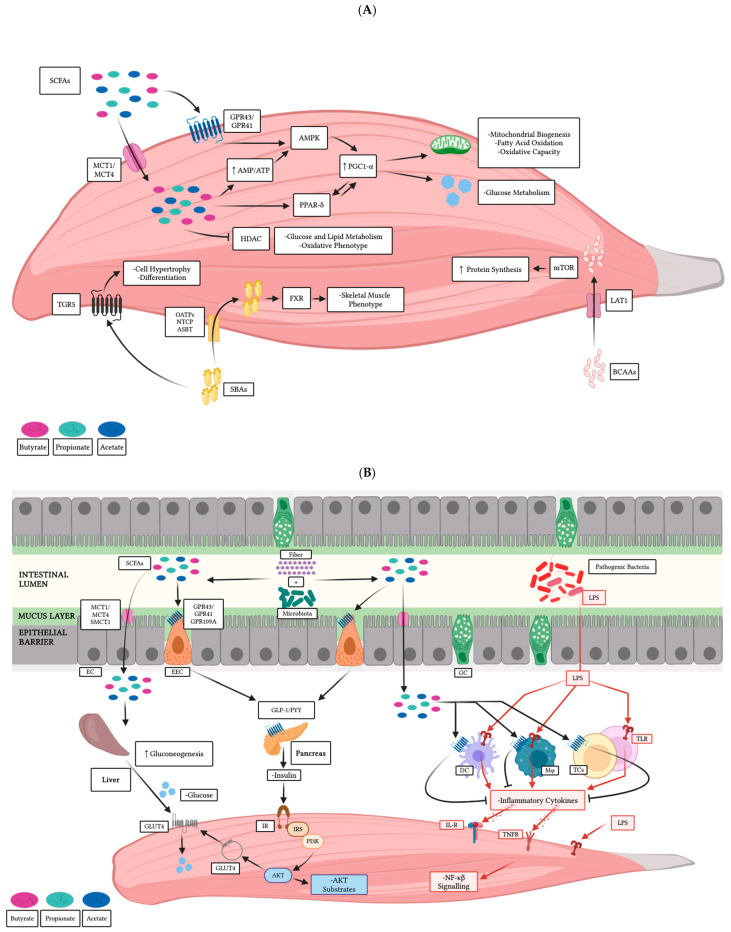
Effects of Secondary Metabolites on Skeletal Muscle. (**A**) SCFAs, SBAs, and BCAAs can directly affect muscle metabolism. SCFAs are mobilized through coupled transporters and activate GPRs on cell membranes, which relay signals intracellularly. Once inside the cell, SCFAs influence components of the AMPK pathway, which are also activated by the aforementioned receptors, as well as PPARs and their coactivator PGC-1α. When activated by certain SCFAs or AMPK, PGC-1α interacts with nuclear receptors, facilitating their recruitment to specific promoters and the assembly of the transcriptional machinery. PPARs function both as nuclear receptors and transcription factors, regulating genes associated with mitochondrial biogenesis, lipid metabolism, and oxidative capacity, thereby promoting the oxidative fiber phenotype. Moreover, in addition to activating nuclear receptors, SCFAs can influence gene expression by interacting with enzymes involved in chromatin condensation. Inhibition of HDACs by these metabolites has been associated with improved glucose and lipid metabolism and an overall oxidative phenotype. SBAs can enter cells through specialized co-transporters and activate nuclear receptors associated with muscle phenotype. These molecules also activate GPRs that promote cell hypertrophy and differentiation. BCAAs can also access the cytoplasm through transporters and ultimately stimulate protein synthesis by activating the mTOR pathway. (**B**) SCFAs can also indirectly influence skeletal muscle metabolism. SCFAs derived from dietary fiber stimulate enteroendocrine cells, via activated GPRs, to secrete hormones such as GLP-1 and PYY, which upregulate insulin synthesis by pancreatic cells. Insulin signaling not only promotes glucose uptake by skeletal muscle cells but also stimulates protein synthesis and reduces proteolysis by activating or inhibiting certain substrates, including mTOR and FOXO transcription factors, respectively. SCFAs, particularly propionate, can promote gluconeogenesis in the liver by serving as a direct substrate, modulating enzymes involved in the pathway, and activating the expression of key genes. The presence of pathogenic bacteria can increase the permeability of the epithelial barrier, allowing for the translocation of molecules such as LPS. This molecule can activate TLRs on immune cells, stimulating the production of pro-inflammatory cytokines. If not counterbalanced by anti-inflammatory signals, these cytokines can affect various tissues and organs. Muscle atrophy has been associated with acute inflammation, where pro-inflammatory cytokines activate the NF-κB pathway, leading to compromised cellular differentiation and proliferation. SCFAs can enhance intestinal barrier integrity and mucin production, while also modulating the inflammatory response mediated by specialized cells, thereby mitigating the effects of inflammation on skeletal muscle. SCFAs: Short-Chain Fatty Acids; MCT: Monocarboxylate Transporter; GPR: G Protein-Coupled Receptor; AMP: Adenosine Monophosphate; AMPK: AMP-Activated Protein Kinase; PPAR-δ: Peroxisome Proliferator-Activated Receptor Delta; PGC1-α: Peroxisome Proliferator-Activated Receptor Gamma Coactivator 1-Alpha; HDAC: Histone Deacetylase; SBAs: Secondary Bile Acids; TGR: Takeda G Protein-Coupled Receptor; OATP: Organic Anion Transporting Polypeptides; NTCP: Sodium Taurocholate Co-transporting Polypeptide; ASBT: Apical Sodium-Dependent Bile Acid Transporter; FXR: Farnesoid X Receptor; BCAAs: Branched-Chain Amino Acids; LAT1: L-Type Amino Acid Transporter 1; mTOR: Mechanistic Target of Rapamycin; SMCT: Sodium-Coupled Monocarboxylate Transporter; EC: Enterocyte Cell; EEC: Enteroendocrine Cell; GC: Goblet Cell; LPS: Lipopolysaccharides; GLP1: Glucagon-Like Peptide 1; PYY: Peptide YY; DC: Dendritic Cell; Mφ: Macrophage; TCs: T Cells; TLR: Toll-Like Receptor; GLUT4: Glucose Transporter Type 4; IR: Insulin Receptor; IRS: Insulin Receptor Substrate; PI3K: Phosphoinositide 3-Kinase; AKT: Protein Kinase B; IL-R: Interleukin Receptor; TNFR: Tumor Necrosis Factor Receptor; NF-κB: Nuclear Factor kappa-light-chain-enhancer of activated B cells. Created in BioRender. Larrea-Álvarez, M. (2025) https://BioRender.com/bakknd0 (accessed on 15 March 2025); Agreement number CY282T7NU5 and QV282T7NZF).

As noted, secondary metabolites are transported via the portal vein to the liver for metabolism. From there, they enter the systemic circulation and distribute to various tissues, including skeletal muscle cells (Figure 3A).

Short-chain fatty acids either activate certain G protein-coupled receptors (GPRs) or are moved into the cell via specialized co-transporters. The uptake of acetate or butyrate activates the AMPK signaling pathway, which can also be stimulated by GPR signaling, stimulating catabolic processes and repressing anabolic ones. AMPK is considered crucial in cellular homeostasis because it becomes activated in response to low energy levels, indicated by a high AMP/ATP ratio [143,144]. Activated AMPK can phosphorylate the PGC-1α, which interacts with transcription factors and nuclear receptors to regulate the expression of genes involved in mitochondrial and glucose metabolism as well as heat production. PGC-1α also serves as a mediator of environmental and physiological stimuli, such as temperature, exercise, and dietary components [145,146]. The associated receptors, PPARs, are expressed in various tissues, including skeletal muscle and myotubes [147,148]. Three isoforms have been identified: PPAR-α, PPAR-β/δ, and PPAR-γ. While PPAR-γ is primarily expressed in adipocytes, PPAR-α and PPAR-β/δ are commonly found in muscle tissue [147,148]. These two receptors are involved in the transcription of genes associated with lipid transport, mitochondrial biogenesis, and fatty acid oxidation. Furthermore, PPAR-β/δ not only influences muscle composition by promoting the formation of oxidative fibers but also improves insulin sensitivity and glucose metabolism [149,150]. Although PPAR-γ is not expressed at the same levels as the other two isoforms, it significantly impacts muscle physiology by regulating muscle regeneration, lipid metabolism, and inflammation [151,152]. Both acetate and butyrate have been linked to the activation of PGC-1α, with butyrate also proving to increase PPAR-δ and PPAR-γ expression in L6 myotubes and skeletal muscle [42,147,153].

Short-chain fatty acids have been observed to interact with and inhibit the action of histone deacetylases (HDACs) (Figure 3A). HDAC4 and HDAC5, for example, not only contribute to modulating myoblast differentiation but also to regulating the oxidation of glucose and lipids [154,155]. HDAC4 and HDAC1 are associated with promoting muscle atrophy in conditions of reduced activity, nutrient scarcity, and innervation absence, while HDAC3 is known to inhibit mitochondrial biogenesis [156,157]. Acetate, propionate, and butyrate are known for their ability to inhibit the action of these deacetylases. In mouse skeletal muscle, butyrate has been shown to reduce the activity of HDACs, leading to the development of an oxidative muscle phenotype. In particular, butyrate reduces the levels of HDAC1, although it has no effect on the expression of HDAC4 [158]. Finally, it has been suggested that AMPK activity may influence HDAC activity. Thus, changes in skeletal muscle physiology and metabolism attributed to this kinase might be associated with HDAC regulation [42].

Compounds generated from bile acid and amino acid metabolism may also influence the physiology of muscle (Figure 3A). The liver secretes primary bile acids, while those metabolized in the intestines by the native microbiota are termed SBAs [52]. Hydrolases and other enzymes involved in the metabolism of these molecules are significantly enriched within the gut microbiome [159]. As these metabolites enter the systemic circulation, they can interact with bile acid receptors on skeletal muscle, thereby influencing lipid metabolism. For example, the two primary secondary bile salts, deoxycholic acid and lithocholic acid, activate the TGR5 receptors. These G-protein-coupled bile acid receptors stimulate muscle cell hypertrophy and differentiation [160,161]. Bile acids and their derivatives play a role in activating the farnesoid X receptor (FXR), which, in turn, promotes the expression of fibroblast growth factor known for its influence on skeletal muscle phenotype. Moreover, the upregulation of this factor also prompts the synthesis of enzymes involved in lipogenesis, thereby contributing to fat accumulation in muscle [53,162]. BCAAs, including leucine, isoleucine, and valine, serve not only as nutrients but also as interactive signaling molecules [54]. These metabolites can access cells via cotransporter proteins and interact with components of the mTOR pathway. Supplementation of these metabolites has been linked to fat deposition and protein synthesis in skeletal muscle [56]. Moreover, the gut microbiota synthesizes the enzymes needed to produce BCAAs, thus contributing to their availability and enhancing their effects [55].

### 4.2. Indirect Effects on Skeletal Muscle

Short-chain fatty acids stimulate the secretion of hormones by specialized cells, which could, in turn, modulate skeletal muscle metabolism (Figure 3B). For example, SCFA-stimulated enteroendocrine cells secrete hormones, such as glucagon-like peptide (GLP-1) and peptide YY (PYY), in response to increased intracellular calcium triggered by GPR receptor signaling [163,164].

Furthermore, it has been suggested that SCFA stimulation may also influence epithelial cell differentiation into L cells, which line the intestinal tract and respond to stimuli by regulating metabolism through the activation of the same receptors [165]. The synthesis of hormones induced by SCFAs can be stimulated not only by GPR activation but also by the ability of these metabolites to inhibit HDAC activity. Indeed, butyrate inhibits them, leading to increased production of PYY by L-cells [166]. These hormones are involved in the complex molecular network that modulates appetite control and food intake. GLP-1 is associated not only with the improvement of glucose absorption and metabolism but also with the enhanced recruitment of skeletal muscle microvasculature [51]. Meanwhile, PYY has been reported to modulate the activity of skeletal muscle in response to metabolic stimuli [167]. GLP-1 and PYY stimulate insulin production by pancreatic cells. Insulin signaling not only affects glucose uptake but also promotes protein synthesis and reduces proteolysis in peripheral organs, such as skeletal muscle [36]. The activated insulin receptor initiates a signaling cascade that involves the stimulation of Akt. This cascade targets downstream factors that promote glucose transporter type 4 (GLUT4) translocation to the cell membrane. Additionally, Akt activates other molecules that facilitate the activation of the mTOR pathway and inactivate those linked to protein degradation, such as GSK-3 and the FOXO transcription factors [36]. Propionate, in particular, can impact the process of gluconeogenesis in the liver by either serving as a direct substrate that enters the tricarboxylic acid cycle or by modulating the function of certain enzymes, including phosphoenolpyruvate carboxykinase (PEPCK) and glucose-6-phosphatase (G6P). Also, propionate influences the expression of crucial genes that facilitate glucose production through the inhibition of histone deacetylases [36].

Systemic inflammation and related health conditions can be triggered by the colonization of pathogenic bacteria and increased permeability of the epithelial barrier, a condition known as “leaky gut” [168]. This condition allows for the passage of harmful molecules (e.g., LPS) into the bloodstream, which are recognized by TLRs on macrophages and dendritic cells (Figure 3B). These immune cells then amplify the response by activating B and T cells. The production of pro-inflammatory cytokines is necessary to initiate and sustain the reaction. However, if these signals are not counterbalanced by anti-inflammatory cytokines, they can enter the bloodstream and affect various tissues and organs [168]. Acute inflammation has been associated with muscle atrophy induced by various pathways, including alterations in protein balance and mitochondrial metabolism [59]. In skeletal muscle, pro-inflammatory cytokines such as the tumor necrosis factor α (TNF-α) can activate the NF-κB pathway, leading to decreased cellular differentiation and proliferation (Figure 3B). Furthermore, IL-1β can stimulate IL-6 expression, which may interfere with insulin receptor substrate 1, thereby restricting the activation of the mTOR pathway and reducing protein synthesis [36].

Short-chain fatty acids are recognized for their influence on the intestinal epithelial barrier function and the host’s immune response. For instance, butyrate has been shown to enhance epithelial permeability by regulating the expression of important proteins such as claudins, zonulin, and occludin, thereby strengthening tight junctions and the overall resilience of the epithelium [169,170]. Moreover, the presence of this fatty acid has not only been demonstrated to elevate the production of *Muc2* but also to enhance the synthesis of antimicrobial peptides such as LL-37, β-defensins, or RegIIIγ in intestinal epithelial cells [171,172]. Moreover, SCFAs can also modulate the expression of cytokines in various cells. For instance, signaling through GPR43 and GPR109a has been observed to prompt the release of IL-18 in intestinal epithelial cells, while butyrate-associated protein acetylation is associated with the downregulation of IL-8 [57,173]. Furthermore, butyrate and propionate are observed to induce the expression of the anti-inflammatory factor TGF1 [174]. These metabolites can also impact gene expression in immune cells, including dendritic cells, macrophages, and lymphocytes. For example, in macrophages, the transcriptional activity of key genes is modulated by butyrate, ultimately decreasing the inflammatory response in cells challenged with LPS. Furthermore, this fatty acid has been associated with the reduction in inflammation in dendritic cells [175,176]. It has been shown that SCFAs can induce the differentiation of Treg cells from their naïve precursors. This process is closely linked to the regulation of Foxp3 expression, a transcription factor essential for T lymphocyte differentiation [177] (Figure 3B). Another type of lymphocyte that is impacted by these metabolites is B cells. Acetate-stimulated GPR43 signaling has been linked to an increase in intestinal IgA levels, and other SCFAs are known to influence plasma cell differentiation from naïve B cells [178,179]. Certainly, enhancing the synthesis of SCFAs to dampen the inflammatory response could serve as a complementary strategy to alleviate the negative effects of inflammation on muscle cells.

## 5. Probiotics, Growth Performance, Muscle Metabolism, and Meat Quality Parameters in Broiler Chickens

Probiotics, in particular, have been utilized to enhance muscle conditions in farm animals and to boost overall performance in farming. Table 1 summarizes certain studies that have highlighted the effects of probiotic exposure on muscle health and meat quality. Broiler chickens have been selectively bred for meat production, with the industry’s efficiency significantly enhanced by advancements in management practices, genetic breeding, nutritional strategies, and disease control methods [180]. Ross 308, Cobb-500, and Arbor Acres are examples of broiler breeds valued in the industry for their growth rates, efficiency in feed conversion, and high (breast) meat yields. Nevertheless, they differ in specific performance parameters and adaptability to different environments. Ross and Cobb are recognized for their superior growth and robustness, with Cobb being the benchmark for fast-growing chickens [181,182]. On the other hand, Arbor Acres are known for their consistent performance and high adaptability to various environments [183]. These strains are chosen based on production goals, environmental conditions, and market demands.

### 5.1. In Ovo Administration

In chickens, muscle hyperplasia primarily takes place during embryonic development, while subsequent muscle fiber growth after hatching is predominantly achieved through hypertrophy [86]. The rationale is that enhancing muscle fiber numbers and establishing supportive vasculature during embryonic development can contribute to improved growth and meat production after hatching [184]. Consequently, in ovo administration of nutrients and other bioactive substances has been investigated not only to support embryonic development and metabolism but also to promote embryonic growth and biogenesis [185,186].

For instance, *L. rhamnosus* NRRL-B-442, along with *L. paracasei* DUP-13076, has been used to promote embryonic growth and muscle development in Ross 308 broilers [187]. In this study, the probiotics were sprayed using an atomizer onto fertilized eggs and incubated. *L. rhamnosus* NRRL-B-442-treated animals showed higher embryo weights compared to the control and *L. paracasei* DUP-13076 groups, while breast and leg weights improved in birds from both experimental groups. Untreated animals exhibited reduced muscle fiber density throughout the entire experiment and demonstrated larger myofiber cross-sectional areas. Probiotic administration also enhanced nuclei density in the pectoralis major muscle compared to the controls. Values were higher for *L. rhamnosus* NRRL-B-442-fed chicks than for *L. paracasei* DUP-13076-fed ones on days 10 and 14, although on day 18, the highest values were observed in animals from the *L. paracasei* DUP-13076 group. The expression of crucial myogenic regulatory factors (*MYF5*, *MYOD*, *MYOG*, and *MRF4*) was reduced by both bacteria on days 10 and 14. In contrast, on day 18, these factors were upregulated by *L. rhamnosus* NRRL-B-442 compared to control conditions, while *L. paracasei* DUP-13076 upregulated *MYF5* and *MYOG* but downregulated *MRF4*. Expression of *IGF1* and its receptor (*IGF1R*) was boosted by both probiotics, although at different time points. However, *L. rhamnosus* NRRL-B-442 induced *IGF1R* downregulation on day 14. *FGF2* and *FGF4* were upregulated by both probiotics on day 18, while *L. paracasei* DUP-13076 reduced transcription of *FGF4* on day 14 [187] (Table 1).

**Table 1:** Effects of probiotics on meat quality parameters, muscle metabolism, and growth performance in broiler chickens.

Broiler Breed	Probiotic Treatment	Administration	Sample	Main Results	Reference
Ross 308	*L. rhamnosus* NRRL-B-442 *L. paracasei* DUP-13076	In ovo spray (∼4 log CFU/egg for 18 days)	Pectoralis muscle	-↑ EW by *L. rhamnosus*; ↑ breast and leg weights by both probiotics -↑ myofiber and nuclei density and ↓ myofiber CSA by both probiotics -↑ expression of *MYF5*, *MYOD*, *MYOG*, and *MRF4* by *L*. *rhamnosus*; -↑ expression of *IGF1, IGF1R*, *FGF2* and *FGF4* by both probiotics	[187]
Cobb-500	*E. faecium* AL41 (CCM 8558)	Orally (1.0 × 10^9^ CFU/0.2 mL of PBS for 7 days)	Pectoralis muscle	-↑ pectoralis major weight and ↑ BWG -↑ total RNA content; no differences in total DNA and total protein content; -↑ transcription levels of *IGF1* and *PAX7*; ↓ transcription levels of *MYF5* -↑ myonuclei number and fiber CSA; ↑ capillary area and capillary density	[188]
Cobb-500	*E. faecium* AL41 (CCM 8558)	Orally (1.0 × 10^9^ CFU/0.2 mL of PBS for 7 days; alone or in combination with *S.* Enteritidis PT4 1.0 × 10^8^ CFU/0.2 mL PBS on day 4)	Pectoralis muscle	-↓ fiber CSA along with ↓ nuclei number and ↓ capillary area caused by *S.* Enteritidis colonization; effects alleviated by the probiotic -↑ CK activity in the presence of *S.* Enteritidis; effects lessened by the probiotic	[189]
Arbor Acres	*L. plantarum* *L. salivarius* *Ligilactobacillus ingluviei* (unspecified strains)	Gavage (10^8^ CFU/mL each alone or in combination (1:1:1) at 1.0 mL/day from 22 to 42 d of age)	Pectoralis muscle	-↑ BW by *L. ingluviei* -↑ myofiber diameter and myofiber density by *L. plantarum* and *L. ingluviei* -↑ relative expression of *MyHC SM*, *MyHC FRM*, *PFK*, *PDH*, *IDH*, *SDH,* and *Tfam* genes by all probiotics; ↑ relative expression of *CoxVa, PK,* and myoglobin genes by *L. plantarum* and *L. ingluviei*	[190]
Ross 308	*L. acidophilus* ATCC 20552	Dietary (3 × 10^8^ CFU/mL and 0.1 g/kg feed from 15 to 37 days of age)	Breast muscle	-↑ BW and breast muscle weight; ↓ FCR -↑ mRNA expression of *IGF1*, *IGF1R* and *GHSR*	[191]
Cobb-500	*L. plantarum* P8	Dietary (1 × 10^8^ CFU/g alone or in combination with 200 μL/day DEX injection 3 mg/kg BW from d 16 to d 21)	Breast muscle	-oxidative stress negatively affected levels of MDA, SOD, GPx, Keap1, Nrf2, mtDNA copy numbers, *PGC-1α*, *SIRT1*, *SIRT3*, *MFF*, *Mfn1,* and *OPA1*; basal levels restored by the probiotic; oxidative stress caused *↓* expression of ATG5, Becline-1, Parkin, PINK1, LC3II/I, LC3B and ↑ levels of NLRP3, IL-18, Caspase-1; basal levels restored by the probiotic	[192]
Arbor Acres	*B. subtilis* DSM32324-32325	Dietary (3.2 × 10^9^ CFU/g at low-dose 300 mg/kg and high-dose 500 mg/kg for 35 d)	Breast and Thigh muscle	-↑ pH_45min_ and pH_24h_ post-mortem in breast and thigh muscle; ↑ redness and -↓ luminance, yellowness, drip loss, cooking loss, and meat shear force -↑ percentage of type I fibers and ↓ percentage of type II fibers in thigh muscle -↑ mRNA levels of *MyHC I* in breast and thigh muscle and ↓ mRNA levels of *MyHC IIb* in breast muscle; no differences in *MyHC IIa*; ↑ slow *MyHC* protein expression and -↓ fast *MyHC* protein expression in thigh muscle; ↑ relative mRNA and protein levels of *AMPK*, *SIRT1*, *PGC-1α*, *CAT*, *SOD*, *GPx*, *Nrf2* and *HO-1* in breast and thigh muscle; -↑ activity of CAT and GPx; ↑ T-AOC and ↓ MDA concentration	[193]
Three-yellow chickens	^1^ EM	Dietary (0.5% EM of 2 × 10^8^ CFU/kg + 3 × 10^7^ CFU/kg alone or in combination with 0.2 mg/kg sodium selenite (S-Se) or 0.2 mg/kg selenium yeast (Y-Se) for 70 d)	Breast and Thigh muscle	-no differences in BW and FCR -↑ pH and muscle color at higher concentrations -negative correlation between shear force and ↓ muscle fiber perimeter, diameter, and CSA; negative correlation between ↑ muscle fiber density and drip loss and meat lightness -↑ mRNA expression of *MYF5, MYOG* and *MRF4* in breast and thigh muscle; ↑ mRNA expression of *MEF2A* and *MEF2D*	[194]

^1^ Effective microorganisms mainly included *Lactobacillus* (unspecified strains) and yeast. EW: embryonic weight; BW: body weight; BWG: body weight gain; FCR: feed conversion ratio; CSA: cross-sectional area; *MYF5*: myogenic factor *5*; *MYOD*: myoblasts determination protein 1; *MYOG*: myogenin; *MRF4*: myogenic regulatory factor 4; *IGF1*: insulin-like growth factor 1; *IGF1R*: insulin-like growth factor 1 receptor; *FGF2*: fibroblast growth factor 2; *FGF4*: fibroblast growth factor 4; *PAX7*: paired box protein 7; CK: creatine kinase; *MyHC SM/MyHC I*: myosin heavy chain slow-twitch; *MyHC FRM/MyHC II*: myosin heavy chain fast-twitch; *PFK*: phosphofructokinase; *PDH*: pyruvate dehydrogenase; *IDH*: isocitrate dehydrogenase; *SDH*: succinate dehydrogenase; *Tfam*: mitochondrial transcription factor A; *CoxVa*: cytochrome oxidase subunits of complex IV; *PK*: pyruvate kinase; *GHSR*: growth hormone secretagogue receptor; DEX: dexamethasone; MDA: malondialdehyde; SOD: superoxide dismutase; Keap1: anti-Kelch-like ECH-associated protein 1; *Nrf2*: anti-nuclear factor-E2-related factor 2; mtDNA: mitochondrial DNA; *PGC-1α*: peroxisome proliferator-activated receptor gamma coactivator 1α; *AMPK*: AMP-activated protein kinase; *SIRT1*: silent information regulator 1; *SIRT3*: silent information regulator 3; *MFF*: mitochondrial fission factor; *Mfn1*: mitochondrial fusion protein 2; *OPA1*: optic atrophy protein 1; ATG5: autophagy-related gene 5; Becline-1: Bcl-2-interacting protein; PINK1: PTEN-induced kinase 1; LC3II/I: light chain 3 II; LC3B: antilight chain 3 B; Parkin: anti-RBR E3 ubiquitin protein ligase; NLRP3: pyrin domain-containing protein 3; IL-18: interleukin 18; Caspase-1: cysteinyl aspartate-specific proteinase-1; CAT: catalase; GPx: glutathione peroxidase; *HO-1*: heme oxygenase 1; T-AOC: total antioxidant capacity; *MEF2A*: myocyte enhancer factor 2A; *MEF2D*: myocyte enhancer factor 2D. Table symbols: ↑ increment; ↓ reduction.

Another study investigated the effects of probiotics combined with prebiotics, administered via injection into Ross 308 eggs [195]. After hatching, the animals were sexed, and the males were housed on an experimental farm before being slaughtered at 35 days of age. Animals treated with *Lactococcus lactis* subsp. *lactis* IBB SL1 and inulin showed higher body weight values compared to the control group and other treatments, which included another probiotic subspecies (*L. lactis* subsp. *cremoris* IBB SC1) and a commercial prebiotic (Bi2tos). These treatments did not lead to changes in daily feed intake, feed conversion ratio, or the percentages of carcass and breast muscle yield. However, the percentage of water-holding capacity was improved by the application of both subspecies and their corresponding prebiotics, although no differences were observed in pH, meat color, or lightness of the pectoral muscle. Birds exposed to *L. lactis* subsp. *lactis* IBB SL1 and inulin exhibited not only a higher percentage of glycolytic fibers but also lower percentages of oxidative fibers in the pectoral muscle compared to animals in the other groups. On the other hand, in ovo injection of *L. salivarius* IBB3154 combined with galactooligosaccharides was shown to upregulate the expression of genes associated with metabolic efficiency, growth, and muscle functionality in the pectoral muscle of 7-day-old Cobb-500FF chickens. These genes included follistatin (*FST*), phosphorylase kinase regulatory subunit beta (*PHKB*), and protein kinase AMP-activated non-catalytic subunit gamma 3 (*PRKAG3*), which contribute to preventing muscle degradation, facilitating glycogen breakdown, and optimizing lipid and carbohydrate metabolism, respectively. However, these effects were not observed in animals at 42 days of age [196]. Overall, in ovo probiotic application was generally found to support embryonic growth and muscle development. Furthermore, treatments exhibited myogenic effects by modulating key genes associated not only with cellular proliferation and differentiation but also with muscle energy metabolism, although limited effects were recorded regarding meat quality parameters.

### 5.2. Orally and Dietary Administration

Satellite cells serve as the primary contributors of new myonuclei, which are incorporated into pre-existing myofibers to support muscle hypertrophy [13,14]. Satellite cell activity and phenotype can be influenced by environmental factors, including nutrition and growth conditions [197,198]. For instance, elevated temperatures and feed deprivation have been shown to impair their mitotic activity, ultimately affecting muscle size [199]. Additionally, infection with *Salmonella* Enteritidis has been reported to disrupt capillarization and reduce myofiber cross-sectional area [189].

Probiotics can be administered orally through methods such as gavage, capsules, or liquid formulations. This approach allows for precise dosage delivery, making it particularly suitable for studies targeting short-term effects or specific conditions such as gastrointestinal infections. However, its practical application in commercial production is limited due to the labor-intensive nature of the process, rendering it less viable for large-scale set-ups [200,201]. *E. faecium* is a bacterial species recognized for its probiotic properties, which include stimulating the immune system and enhancing the integrity of the intestinal barrier [202,203]. It has been tested in Cobb-500 broilers to assess its impact on muscle development and growth performance [188]. In particular, *E. faecium* AL41 administration enhanced body weight gain and pectoralis muscle weight. Samples from the probiotic-treated group exhibited a higher total RNA content compared to controls, although no significant differences were observed in total DNA and protein content. Furthermore, muscle samples from treated chickens demonstrated larger fiber cross-sectional areas and an increased number of myonuclei per fiber, indicating enhanced muscle development. Likewise, capillarization was positively influenced in the treated chicks, evidenced by an increase in capillary supply and an enlarged area covered by capillaries. Gene expression analyses demonstrated enhanced transcription levels of both *IGF1* and *PAX7* and decreased levels of *MYF5* in the pectoralis muscle of *E. faecium* AL41-supplemented birds (Table 1).

*E. faecium* AL41 has also been used to modulate the effects of *S.* Enteritidis PT4 colonization on the muscle tissue of Cobb-500 broilers (Table 1) [189]. This intracellular pathogen is responsible not only for reducing the welfare of animals but also for important production losses while increasing the potential contamination of poultry products [204]. They attach to villi, enter epithelial cells, and multiply. This intestinal damage leads to complications in digestion and nutrient absorption and triggers inflammation, which negatively impacts skeletal muscle development [205,206]. In control animals, a time-dependent hypertrophic growth of myofibers in the pectoralis muscle was observed, which was suppressed by bacterial treatment, including both probiotics and the pathogen. However, *E. faecium* AL41 compensated for the reduced number of nuclei and limited fiber cross-sectional area detected after *S.* Enteritidis PT4 colonization. In addition, this serovar negatively affected muscle capillarization, impacting oxygen delivery and nutrient translocation. The probiotic not only alleviated this outcome but also lessened the upregulation of CK activity. This enzyme, indicative of cellular stress, had its concentration and activity increased in the presence of *S.* Enteritidis PT4.

The effects of *L. plantarum*, *L. salivarius*, and *L. ingluviei* (unspecified strains), delivered via gavage, on pectoralis muscle characteristics were evaluated in Arbor Acres broiler chickens [190]. In 42-day-old birds, body weight was uniquely enhanced by *L. ingluviei*, which, along with *L. plantarum*, improved myofiber diameter and density. All bacterial species increased the relative expression of myosin heavy chain (MyHC) genes associated with red fast- and slow-twitch myofibers, *MyHC FRM,* and *MyHC SM*, respectively. They also upregulated genes involved in energy metabolism pathways, including phosphofructokinase *(PFK)*, pyruvate dehydrogenase *(PDH)*, isocitrate dehydrogenase *(IDH)*, succinate dehydrogenase *(SDH)*, as well as mitochondrial transcription factor A *(Tfam)*, which is critical for maintaining mitochondrial integrity and function. Furthermore, *L. plantarum* and *L. ingluviei* induced increased transcription of genes encoding cytochrome oxidase subunits of complex IV (*CoxVa*), pyruvate kinase (*PK*), and myoglobin [190] (Table 1). In general, oral administration of probiotics has been shown to enhance myofiber growth by improving capillarization and regulating glucose metabolism. Also, probiotics may have protective effects against bacterial infections that can negatively impact growth performance and meat quality.

Probiotics can also be administered through dietary inclusion, either by incorporating them into feed or water. This approach ensures consistent exposure, as animals gradually consume the probiotics during regular feeding, resulting in cumulative effects not only on gut health through interactions with the gut microbiota but also on immune modulation. Furthermore, dietary administration is practical for commercial farming as it seamlessly integrates into standard farming practices [207,208]. For instance, dietary supplementation with *L. acidophilus* ATCC 20552 has been studied for its effects on growth performance and muscle gene expression in Ross 308 broilers. Treated birds exhibited a reduced feed conversion ratio, along with increased body weight and breast muscle weight, compared to control birds. In addition, the mRNA abundance of *IGF1*, *IGF1R*, and the growth hormone secretagogue receptor (*GHSR*), which stimulates growth hormone secretion, was significantly higher in the supplemented group (Table 1) [191]. On the other hand, the administration of *L. fermentum* 2i3, either alone or in combination with humic acids via drinking water, did not result in significant changes in growth parameters such as body weight and feed consumption. However, the probiotic positively influenced immune responses, including increased IgA production, enhanced phagocytic activity, and modulation of T-lymphocyte subpopulations. Despite these immunological benefits, the administration of *L. fermentum* 2i3 did not affect the expression of factors such as *MYF-5*, *IGF-2*, and *PAX7*. Interestingly, the abundance of *PAX7* was positively influenced by exposure to humic acids [209].

Oxidative stress caused by intensive feeding has been reported in broiler chickens, leading to lipidosis, fibrosis, and myodegeneration, which adversely affect meat quality parameters such as color and nutritional value [210]. For instance, birds exposed to dexamethasone (DEX)—a synthetic glucocorticoid commonly used in oxidative stress models—produced meat of lower quality compared to those under control conditions [211]. *L. plantarum* P8, a probiotic strain known for enhancing growth performance and meat quality in chickens [212], has been evaluated for its effects on the antioxidant capacity and inflammation of breast meat in Cobb-500 broilers (Table 1). The probiotic treatment restored basal levels of malondialdehyde (MDA)—a marker of lipid peroxidation—and the mRNA expression and enzymatic activity of superoxide dismutase (SOD) and glutathione peroxidase (GPx), which were negatively affected by oxidative stress. A similar trend was observed in the protein expression of kelch-like ECH-associated protein 1 (Keap1) and the nuclear factor erythroid 2-related factor 2 (Nrf2), which are regulators of the oxidative defense. In addition, the relative copy number of mtDNA, which decreased in DEX-exposed animals, was restored by *L. plantarum* P8. The probiotic also reestablished the mRNA abundance of not only *PGC-1α*, *SIRT1*, and *SIRT3* but also the mitochondrial fission factor (*MFF*), mitofusion 1 (*Mfn1*), and optic atrophy 1 (*OPA1*), all of which are crucial for mitochondrial function. Furthermore, *L. plantarum* P8 enhanced the expression of factors involved in mitophagy, including the autophagy-related gene 5 (ATG5), beclin-1, parkin, PTEN-induced kinase 1 (PINK1), and the microtubule-associated protein 1A/1B-light chain 3 II/I (LC3II/I) and B (LC3B), which were upregulated following DEX injection. Animals in the DEX-treated group exhibited elevated expression of the NOD-like receptor family pyrin domain containing 3 (NLRP3), IL-8, and Caspase-1, markers of inflammasome activation and pro-inflammatory cytokines. These levels were significantly reduced by P8 administration, suggesting that the probiotic’s ability to attenuate inflammasome activation and the associated inflammatory response may be linked to its enhancement of mitophagy [192]. In general, members of the Lactobacillaceae family have been shown to not only positively influence growth performance but also alleviate the effects of oxidative stress in the breast meat of broilers.

*B. subtilis* has been utilized in farm animals, including broiler chickens, to evaluate physiological responses and performance parameters [213,214]. *B. subtilis* DSM32324-32325 has also been investigated for its probiotic effects on meat quality, as well as its ability to influence muscle fiber type transformation and muscle antioxidant activity in Arbor Acres broilers [193] (Table 1). Administration of this bacterial strain resulted in increased pH values at both 45 min (pH_45min_) and 24 h (pH_24h_) post-mortem in the thigh and breast muscles. Moreover, the redness values of the meat were elevated, while the yellowness and luminescence values, along with drip loss percentages, were reduced. Similarly, cooking loss and meat shear force were decreased in the presence of the probiotic. In the breast muscle, the composition was exclusively of type II fibers, which remained unaffected by probiotic treatment. However, in the thigh muscle, *B. subtilis* DSM32324-32325 administration led to a higher percentage of type I fibers and a corresponding reduction in type II fibers compared to the control group. The treatment also increased mRNA levels of *MyHC I* in the thigh muscle while reducing those of *MyHC IIb* in the breast muscle; no significant differences were observed in the expression of *MyHC IIa*. Furthermore, in the thigh muscle, the probiotic enhanced the expression of slow *MyHC* protein and decreased that of the fast *MyHC* protein. To investigate the underlying mechanisms of these effects on muscle metabolism, the expression levels of key regulatory proteins were analyzed. The relative mRNA and protein levels of *AMPK*, *SIRT1*, and *PGC1-α* were significantly elevated following *B. subtilis* DSM32324-32325 administration. Antioxidant-related indicators further demonstrated the probiotic’s potential to mitigate oxidative stress. Both mRNA and protein expression levels of catalase (CAT), SOD, and GPx, as well as Nrf2 and the heme oxygenase 1 (HO-1), were upregulated in the breast and thigh muscles of *B. subtilis* DSM32324-32325-fed broilers. Nrf2 regulates the expression of antioxidant enzymes, while HO-1 is associated with the production of potent antioxidant scavengers, such as bilirubin, which play a critical role in oxidative defense. Correspondingly, the activities of CAT and GPx were enhanced, along with total antioxidant capacity, while the concentration of MDA decreased in the experimental groups compared to the controls [193].

Another strain of *B. subtilis*, PB6, was investigated for its effects on meat quality parameters in the breast and thigh muscles of Ross 708 broiler chickens subjected to preslaughter stress [215]. Dietary probiotic treatment not only reduced the pH of the breast and thigh muscles but also decreased cooking loss while improving meat lightness, redness, and yellowness, along with enhanced water-holding capacity. Antioxidant analysis of the breast muscle revealed that probiotic administration increased total antioxidant capacity and reduced protein carbonyl concentrations, although no significant changes in MDA levels were observed [215]. On the other hand, a different species, *B. licheniformis* DSM 28710, has been used to ferment wet feed to evaluate its effects on growth efficiency and the expression of key muscle-related genes in the breast muscle of Cobb-500 broilers [216]. Fermentation of wet feed has been shown to improve the digestibility of organic acids and crude proteins [217]. Probiotic fermentation is, therefore, considered beneficial due to its positive contributions to animal physiology. In broilers fed probiotic-fermented wet feed, the final body weight was significantly higher compared to those in the control groups, and the feed conversion ratio was reduced. Furthermore, the expression levels of *IGF-1*, myostatin, growth hormone, and mTOR were comparable to those in the dry-feed control group but higher than in the wet-feed group without probiotic fermentation [216]. Notably, *B. subtilis* strains have been shown to improve muscle oxidative responses, modulate fiber composition, and enhance meat quality parameters in broilers.

A strain of *E. faecium* CGMCC 4847, along with *C. butyricum* B1, was evaluated for its dietary effects on growth performance, meat quality parameters, and lipid metabolism in the breast and thigh muscles of Ross 308 broiler chickens [218]. *C. butyricum* B1 positively influenced average daily gain and average daily feed intake while also increasing the intramuscular fat content in both breast and thigh muscles. Furthermore, it enhanced the activity of lipoprotein lipase (LPL) in breast muscle and enhanced the relative mRNA abundance of the *LPL* gene. Elevated mRNA levels of the *LPL* gene have been detected in the adductor superficialis of chickens, a predominantly oxidative twitch muscle, as this enzyme hydrolyzes circulating lipoproteins to release free fatty acids that support oxidative metabolism [219]. *C. butyricum B1* reduced the relative abundance of *Bacteroidetes* without affecting *Firmicutes* in the cecal contents, whereas *E. faecium* CGMCC 4847 administration showed no significant effects [218]. However, another strain of *E. faecium*, CGMCC 2516, included in the diet of Arbor Acres chickens, demonstrated beneficial effects on improving the meat quality of broilers by modulating the abundance of key proteins involved in muscle metabolism [220]. The carcass percentage of leg and pectoral muscle was enhanced by probiotic treatment. Certain meat quality parameters of the latter were also improved, including water holding capacity, pH, and color. *E. faecium* CGMCC 2516 prompted the differential expression of 22 proteins, 17 of which were associated not only with carbohydrate metabolism but also with tight junction pathways. Phosphoglucomutase-1 and fructose 1,6-bisphosphatase upregulation suggested a more efficient storage of energy substrates, whereas the upregulation of glycolytic enzymes hinted at a delayed post-mortem glycolytic activity, slower ATP depletion, and reduced myofibril degradation, all of which are related to tender meat. On the other hand, the enhanced water-holding capacity and pH values were linked to the downregulation of β-enolase and pyruvate kinase muscle isozyme, which lessened the conversion of pyruvate into lactic acid; upregulation of CK also contributed to muscle moisture. Tenderness has been associated with cellular homeostasis; and *E. faecium* CGMCC 2516 induced the upregulation of heat-shock proteins, which may help maintain muscle integrity and protein repair, directly influencing meat characteristics [220].

The commercial probiotics Biosol, containing *E. faecium* (unspecified strain) along with dextrose, betaine, lactose, and Zemos, are composed of *B. subtilis*, *B. amyloliquefaciens*, and *L. acidophilus* (unspecified strains) and, combined with humic and citric acid, have been shown to improve growth performance and muscle metabolism in Cobb-500 broilers. Specifically, the administration of these products through drinking water enhanced body weight and feed conversion ratio. Furthermore, the breast and thigh muscle weights were significantly higher in birds treated with Biosol or a combination of both products compared to the control group. Moreover, birds in the experimental groups exhibited upregulation of the SET and MYND domain containing-1 (*Smyd1*) involved in myofibril assembly and muscle gene expression—*mTOR*, and *TLR-4*—alongside downregulation of *NBN* (Nibrin), associated with DNA repair in breast and thigh muscles. Biosol supplementation increased cecal *Lactobacilli* counts, reduced *E. coli* levels, and lowered the aerobic bacterial load [221]. Another study investigated the effects of dietary administration of a probiotic combination known as effective microorganism (EM), comprising *Lactobacillus* (unspecified strains) and yeast, on muscle fiber characteristics, meat quality parameters, and growth performance in Three-yellow chickens, a representative breed in China [194] (Table 1). While EM administration did not affect growth performance parameters such as body weight and feed conversion ratio, it increased muscle pH and improved muscle color at higher concentrations compared to lower doses. The treatment resulted in a reduction in muscle fiber perimeter, diameter, and cross-sectional area, which were negatively correlated with shear force. Conversely, muscle fiber density was increased and found to be negatively correlated with drip loss and meat lightness. The expression of myogenic regulatory factors, including *MYF5*, *MYOG*, and *MRF4*, was elevated in the breast and thigh muscles of birds exposed to EM, correlating with the observed increase in muscle density. Finally, EM treatment enhanced the levels of the transcription factors *MEF2A* and *MEF2D*, which are critical for muscle development, differentiation, and the formation of slow muscle fibers [194]. Overall, the use of combined microorganisms may enhance growth performance and meat quality by influencing muscle fiber characteristics and the expression of muscle-associated genes.

Various species of the fungus *Aspergillus* have been extensively utilized in food processing and industrial enzyme production. Some of these species have been granted GRAS (Generally Recognized as Safe) status by the Food and Drug administration (FDA) [222] and have consequently been investigated for their probiotic potential [135]. In particular, *A. awamori* (unspecified strain) has been evaluated for its properties, focusing on its effects on growth performance and the expression of genes involved in skeletal muscle protein metabolism in Chunky broiler chickens [223]. Animals fed a basal diet supplemented with *A. awamori* exhibited a reduced feed conversion ratio compared to control conditions, as body weight gain increased while feed intake decreased. Moreover, breast muscle weight was enhanced in these animals. The mRNA expression of genes associated with protein turnover, including ubiquitin, proteasome C2, and μ-calpain, was elevated; however, no differences were observed in the expression levels of atrogin-1 and m-calpain. Similarly, transcript abundance of myosin and PAX7 remained unchanged by the probiotic treatment, whereas β-actin expression was upregulated [223]. This fungal species has also been evaluated for its effects on similar parameters in Lohmann broilers [224]. Birds exposed to *A. awamori* (unspecified strain) demonstrated improved feed intake and feed conversion ratios, with these effects further enhanced by the addition of the prebiotic fructooligosaccharide. Also, the mRNA expression levels of key factors involved in muscle growth, repair, and metabolic efficiency, including *IGF1*, *IGF1R*, and *GHSR*, were significantly elevated in treated birds compared to those of the control group [224].

Through metabolite production and gut microbiome modulation, probiotics influence muscle metabolism, favoring oxidative fiber characteristics associated with enhanced meat quality. As discussed, probiotic metabolites can improve mitochondrial biogenesis and oxidative phosphorylation via the AMPK-PGC-1α pathway, promoting oxidative muscle metabolism. This not only helps maintain post-mortem muscle pH and reduce drip loss but also lowers shear force, as the oxidative phenotype is characterized by type I fibers. Furthermore, higher myoglobin levels enhance meat color, while modulation of key genes could improve myofiber density, structure, and tenderness.

Evaluating the three probiotic administration routes reveals both common benefits and distinct impacts on muscle biology in broiler chickens. In ovo supplementation, applied pre-hatch, primarily enhances early muscle formation—improving body weight, muscle mass, oxidative fiber proportions, and upregulating key myogenic regulators and growth signaling genes, which, in turn, improves water-holding capacity. In contrast, per os administration, provided post-hatch, mainly boosts pectoralis muscle weight and improves fiber characteristics such as cross-sectional area, capillarization, and myonuclear density, along with increased expression of myogenic factors and energy metabolism enzymes. Dietary supplementation, also administered post-hatch, yields the most comprehensive outcomes by significantly enhancing overall growth performance, muscle fiber dimensions, and meat quality through broad modulation of signaling pathways, myogenesis, oxidative metabolism, and other metabolic processes. Overall, while all approaches offer positive impacts, in ovo treatment targets early developmental processes, whereas per os and dietary strategies primarily enhance post-hatch muscle growth—with dietary supplementation providing the most extensive benefits.

Other strategies, such as prebiotics, synbiotics, or phytobiotics, have also been tested. Prebiotics are non-digestible fibers, primarily polysaccharides, that cannot be broken down by monogastric animals but selectively stimulate the growth and activity of beneficial gut bacteria. In contrast, synbiotics combine prebiotics with probiotic bacteria to enhance gut health [225]. The administration of prebiotics and synbiotics has been linked to improved intestinal health, immune function, and growth performance in broilers [226,227,228,229]. In ovo application of galactooligosaccharides (GOS) did not affect fiber diameter but mitigated the effects of histopathological conditions in breast muscle [230]. Conversely, chitin oligosaccharides reduced shear force by inducing an oxidative phenotype, increasing the expression of MyHCI, MYOD, and SIRT1, as well as antioxidant enzymes such as CAT, SOD, GPx, and HO-1 [231]. Moreover, dietary supplementation with *Enteromorpha* polysaccharides increased breast yield and upregulated genes involved in signaling pathways and immune response [232]. The combination of *L. lactis* subsp. *lactis* IBB SL1 and inulin enhanced body weight and improved water-holding capacity [195], whereas the mixture of *L. lactis* subsp. *cremoris* IBB477 and GOS did not produce any noticeable effects [230].

Phytobiotic supplementation has also been shown to positively impact gut health, growth performance, and meat quality in broiler chickens [233,234]. Phytobiotics comprise biologically active compounds derived from plants or algae, such as polyphenols, flavonoids, and essential oils, which can promote various health benefits in animals [235]. Dietary administration of chicory, St. John’s wort, safflower, and creeping thyme was shown to improve body weight and modulate the expression of MYOD and myostatin [236]. The abundance of these myogenic factors was also regulated by a formulation containing spice extracts, herbs, and vegetable oils, which also influenced the expression of inflammatory markers IL-6 and TNF-α [237]. Moreover, the dietary addition of amino acids (e.g., methionine, arginine) has been shown to regulate the expression of myogenic factors (MYOD, MYOG, Myf5, MRF4) and positively influence post-mortem pH and meat color [238,239,240]. Overall, these supplementation strategies share some benefits with probiotics, such as promoting oxidative fiber enrichment and improving shear force. Furthermore, phytobiotics and amino acids enhance performance metrics and meat quality traits, including pH, lipid content, and water-holding capacity. However, probiotics—particularly dietary and in ovo administration—have the most comprehensive impact on muscle development, metabolism, and meat quality in broilers. They uniquely regulate myogenic factors, key signaling pathways (IGF-1, AMPK, PGC-1α), and oxidative metabolism, resulting in enhanced myofiber structure and post-mortem muscle properties.

Despite the positive outcomes observed, several challenges hinder the widespread use of probiotics for sustainable and efficient production. These include strain selection and specificity, optimal dosage and administration, mode of delivery, microbiome interactions and mechanisms, and cost-effectiveness and commercial viability [241,242]. Consequently, further research must focus on the optimal timing and administration methods, as well as the potential synergistic effects of combining in ovo and sustained dietary supplementation. In addition, future studies should not only focus on transcriptomic and metabolomic analyses to identify strain-specific metabolic pathways that enhance myogenesis and oxidative muscle phenotype but also examine potential epigenetic modifications regulating muscle proteins, which could have long-term effects. Research in broiler chickens should also explore how probiotics influence host–microbiome crosstalk related to lipid metabolism and its impact on intramuscular fat deposition. Finally, evaluating the cost-effectiveness and scalability of probiotic interventions is essential for their widespread implementation. The cost-effectiveness and scalability of probiotic interventions should also be evaluated to promote widespread implementation in the industry.

## 6. Conclusions

The studies summarized herein show the potential benefits of probiotic administration on broiler muscle characteristics, meat quality, as well as growth performance parameters. Overall, probiotic treatment has been shown to modulate the expression of not only myogenic factors but also genes encoding proteins and enzymes involved in signaling pathways, carbon metabolism, inflammatory response, mitochondrial dynamics, and antioxidant activity. The regulation of these factors has been linked to improvements in meat quality parameters, including pH, color, water-holding capacity, and shear force, as well as enhanced growth performance indicators such as body weight and feed conversion ratio. Recently, public concerns over antibiotic application in animal husbandry have led various countries to ban their use as growth promoters. The use of antibiotics has been a crucial component of the industry’s strategy for management, breeding, and disease control in broiler chickens. Consequently, complementary approaches must be explored, not only to mitigate the effects of infectious diseases but also to sustain production while enhancing muscle characteristics and meat quality parameters. Probiotics have been proposed as a means to positively modulate the gut microbiota, not only preserving intestinal homeostasis but also influencing the production of secondary metabolites, which can directly or indirectly affect skeletal muscle metabolism by regulating the expression and activity of receptors and signaling molecules associated with key muscle-related genes. In the absence of antibiotics, probiotic bacteria represent a viable option for supporting broiler chicken growth and muscle phenotype. A variety of strains, administered either in ovo, orally, or through dietary supplementation, have shown promising results with regard to muscle metabolism. Further research, nonetheless, should also focus on deciphering the cellular and molecular mechanisms behind these outcomes, which could contribute to the identification of new therapeutic targets. Certainly, the evidence collected to this point shows that probiotics should be contemplated as beneficial ingredients of nutritional supplements focusing on muscle metabolism and the quality of meat.

## Data Availability

No new data were created or analyzed in this study.
